# Insights into the Role of MicroRNAs in the Onset and Development of Diabetic Neuropathy

**DOI:** 10.3390/ijms20184627

**Published:** 2019-09-18

**Authors:** Raffaele Simeoli, Alessandra Fierabracci

**Affiliations:** Infectivology and Clinical Trials Area, Bambino Gesù Children’s Hospital, IRCCS, Viale San Paolo 15, 00146 Rome, Italy; raffaele.simeoli@opbg.net

**Keywords:** diabetes mellitus, diabetes complications, diabetes neuropathy, microRNAs, peripheral nervous system, central nervous system

## Abstract

Diabetic neuropathy is a serious complication of chronic hyperglycemia in diabetes patients. This complication can involve both peripheral sensorimotor and autonomic nervous system. The precise nature of injury to the peripheral nerves mediated by chronic hyperglycemia is unknown; however, several mechanisms have been proposed including polyol pathway activation, enhanced glycation of proteins and lipids, increased oxidative stress, and cytokine release in the site of injury. MicroRNAs (miRNAs) are small non-coding RNAs that mediate RNA interference by post-transcriptionally modulating gene expression and protein synthesis. Therefore, they have been implicated in several developmental, physiological, and pathophysiological processes where they modulate the expression of different proteins. Recently, miRNAs gained an increasing attention also for their role as diagnostic test in many diseases due to their stability in serum and their easy detection. Furthermore, recent studies suggest that miRNAs may be involved in diabetic neuropathy although their role in the onset and the development of this complication is not fully understood. In this review, we discuss the most recent literature providing evidence for miRNAs role in diabetic neuropathy opening new pathways to improve both early diagnosis and treatment of this complication.

## 1. Types of Diabetes

Diabetes mellitus (DM) is a chronic metabolic disease whose incidence worldwide is increasing; overall, about 100 million people are affected. Classically, two forms of DM are distinguished. Type 1 diabetes (T1D), defined also as insulin-dependent diabetes mellitus, is a multifactorial disorder characterized by the organ-specific autoimmune destruction of pancreatic insulin producing beta cells in human leukocyte antigen (*HLA*) genetically predisposed subjects [1]. This form occurs in 5%–10% of DM patients [1]. T1D derives from a breakdown in immune regulation that leads to expansion of autoreactive CD4+ and CD8+ T cells, autoantibody-producing B-lymphocytes and activation of the innate immune system [1]. The presence of a chronic inflammatory infiltrate within pancreatic islets leading to insulitis is the main histopathological finding in T1D [2]. Another important aspect is that, in patients with longstanding disease, the remaining pancreatic β-cells resistant to autoimmune destruction are incapable of regeneration [3]. Nevertheless, in contrast to adolescents and adults, recent studies confirm that β-cell regeneration occurs in infants and very young children [3,4].

Type 2 diabetes (T2D), caused by altered insulin function [5], and also defined as non-insulin-dependent diabetes mellitus occurs in 90%–95% of DM patients. In T2D there is a normal production of insulin from pancreatic β-cells; however, peripheral tissues show resistance to the insulin-mediated action resulting in pathological glucose intolerance. In terms of pathogenesis, nowadays, T2D is considered the result of both genetic predisposition and environmental factors such as high calorie diet, low energy expenditure, and generally a more modern lifestyle. β-cell loss and dysfunction through genetic or cytotoxic factors is also predisposing to glucose intolerance together with insulin resistance (IR) in target tissues (American Diabetes Association, ADA 2014) [5]. IR is a pathological condition in which insulin-dependent cells do not respond to normal circulating levels of insulin [6]. Since insulin mediates the entry of glucose into cells, any alteration of its transduction pathway leads to hyperglycemia due to inability of target cells to uptake glucose [6]. However, insulin signal transduction is complex and involves many enzymes and modulatory proteins, therefore an impairment of its signaling may happen at different levels making the exact pathophysiology of IR unclear [6]. Oxidative stress, inflammation, insulin receptor mutations, endoplasmic reticulum stress, and mitochondrial dysfunctions all contribute to IR [6].

In this review, we discuss the most recent literature on diabetes neuropathy. In particular, we aim to underline the implications of miRNAs in the development of this complication and their potential role as biomarkers and therapeutic targets for future treatments.

## 2. Diabetes Complications

In both types of diabetes, deregulation of glucose metabolism is accompanied by characteristic long-term degenerative effects [7]. Commonly, the complications of chronic hyperglycemia are divided into macrovascular complications including endothelial dysfunction (ED) and cardiovascular diseases (CVD); microvascular complications of diabetic retinopathy, nephropathy, and peripheral neuropathy (PN) [7].

CVD is the major cause of morbidity and mortality in patients with DM. Cardiac risk factors, including hypertension, dyslipidemia, smoking, genetic factors, hyperglycemia, insulin resistance/hyperinsulinemia contribute to the pathogenesis [8]. Cardiovascular dysfunction during deregulated glucose metabolism is associated with alterations in factors that modulate vascular function such as nitric oxide (NO), endothelin-1, and endothelial growth factors involved in angiogenesis [9]. Cardiac autonomic neuropathy is manifested as tachycardia and postural hypotension [7]. Cardiovascular autonomic dysfunction is associated with increased risk of silent myocardial ischemia and mortality.

Diabetic nephropathy, leading to chronic kidney disease and renal failure, is characterized by altered urinary albumin excretion, glomerular lesions, and loss of glomerular filtration rate [10]. Nephropathy, strictly related to hyperglycemia, develops in only 35–45 percent of patients with T1D and less than 20 percent of those with T2D [11,12]. Hyperglycemia induces renal damage directly or through hemodynamic modifications such as glomerular hyperfiltration, shear stress, and microalbuminuria contributing to abnormal stimulation of resident renal cells that produce more transforming growth factor (TGF)-β1 [13]. This cytokine causes augmented extracellular matrix protein deposition at the glomerular level, mesangial expansion, and glomerular basement membrane thickening [13].

Retinopathy is the most common microvascular complication of diabetes. Around 10,000 new cases of blindness are reported every year in the United States [14]. Development of retinopathy in T2D is related to both severity of hyperglycemia and presence of hypertension; many T1D patients develop symptoms of retinopathy within 20 years of diagnosis [15]. Background retinopathy is characterized by small hemorrhages in the middle layers of the retina, microaneurysms, retinal edema, and proliferative retinopathy by the formation of new retinal blood vessels leading to vitreous hemorrhage [16]. Osmotic and oxidative stress due to the hyperglycemia may play an important role in cellular injury. 

## 3. MicroRNAs and Diabetic Neuropathy

### 3.1. Diabetic Neuropathy

Diabetic neuropathy (DN) is the most common and troublesome DM complication. ADA defines DN as “the presence of symptoms and/or signs of peripheral nerve dysfunction in people with diabetes after the exclusion of other causes”. The exact prevalence is unknown varying from 10% to 90% of reported cases according to definition criteria [17]. DN can involve both peripheral sensorimotor and autonomic systems, although other forms include cranial and peripheral motor neuropathies [7]. Similarly to microvascular complications, duration of hyperglycemia influences the possibility of developing DN [16]. Although neuropathies appear a consequence of long-lasting diabetes, early-onset polyneuropathy has been reported [18]. Electro-physiologic studies have demonstrated slowed motor and sensory nerve conduction in several patients after 5–10 years of diabetes [19,20]. 

Diabetic patients affected by PN may manifest sensory, focal/multifocal, and autonomic neuropathies. Since high morbidity and mortality are associated with DN, its early diagnosis is critical for prevention and treatment.

Chronic sensorimotor distal symmetric polyneuropathy (DSPN) is the most common form of DN [16]. The main symptom is paresthesia that progresses to hypoesthesia and is manifested as reduction or absence of reflexes, and decreased sensation to vibration and light touch [21]. Typically, patients experience burning, tingling, pain that feels like a shock and sometimes simple numbness that is worse at night [7]. Loss of sensation in the feet and altered foot morphology that is asymptomatic can result in foot ulceration, which is the principal risk of PN; it is important to realize that lack of symptoms does not exclude presence of neuropathy [7]. In fact, more than 80% of amputations occur after a foot ulceration or injury, which goes untreated because the patient has distal neuropathy [22]. Some patients have a painful PN characterized by dysesthesia with lancinating or burning pain that in the worst case leads to anorexia and depression [17]. Focal motor (cranial and peripheral) and compression neuropathies are less common than sensorimotor neuropathies. They are accompanied by cranial or peripheral nerve palsy such as carpal tunnel syndrome and foot drop, and the symptoms include weakness or loss of sensation in nerve distribution [7]. Usually, these neuropathies improve spontaneously in six weeks to six months and only in cases of nerve compression lesions, a surgical intervention of decompression is adopted [7].

Gastrointestinal symptoms can be detected within 5–10 years after the onset of T1D although clinical autonomic neuropathies are less frequent [7]. Autonomic neuropathies affecting gastrointestinal tract comprise gastroparesis (early satiety, nausea, vomiting) and diarrhea (nocturnal with incontinence) [23]. Meanwhile genitourinary neuropathies are characterized by impotence, retrograde ejaculation, and overflow incontinence [23]. Treatment of autonomic neuropathy in diabetics is targeted toward the organ system that is affected, but also includes optimization of glycemic control. Except for the sensorimotor neuropathy that may lead to diabetic foot, there are no available treatments apart from foot care to prevent trauma and ulcers [21].

### 3.2. Functions of MicroRNAs

MicroRNAs (miRNAs, miRs) are single-stranded, small noncoding RNAs of 19–25 nucleotides that mediate RNA interference (RNAi) by post-transcriptional gene silencing [24]. Thus, the miRNAs are involved in several developmental, physiological, and pathophysiological processes where they alter and modulate the expression of different proteins [25]. miRNAs act through two distinct mechanisms sequence-dependent: cleavage of their respective target mRNA (perfect complementary mechanism) or by inhibiting gene translation after complete or only partial binding to their target sequence (imperfect complementary mechanism), respectively [26]. Each miRNA may target many different genes, and many genes can be regulated by different miRNAs [27,28]. Recently, miRNAs have obtained an increasing attention as potential useful diagnostic test in many diseases thanks to their stability in serum and their easy detection [29]. Therefore, circulating miRNAs have been already considered as predictive biomarkers for aging [30], cancer [29], and neurological disorders such as Alzheimer’s disease (AD) [31], multiple sclerosis [32], Parkinson’s disease (PD) [33], and epilepsy [34]. In addition, miRNAs have been also proposed as potential biomarkers for diabetes and its vascular complications [35]. Indeed, alterations of specific miRNA levels can lead not only to the development of chronic inflammation observed in diabetic patients but also to β-cell loss/dysfunction and dysregulation of insulin secretion and signaling along with insulin-resistance and its chronic complications [36]. Therefore, the development of specific antagonists that inhibit specific miRNAs may offer new therapeutic options for diabetes complications.

### 3.3. Role of MiRNAs in the Nervous System

miRNAs play an important role in neurogenesis, neuron survival, dendritic outgrowth, and spine formation [37]. Accordingly, animal models in which *Dicer*, the enzyme responsible for the cleavage of the pre-miRNA into mature miRNAs, has been knocked down in different brain areas leading to apoptosis and neuronal degeneration [38,39]. Furthermore, dysregulation of miRNAs affect the expression of specific proteins associated with neurodegenerative diseases such as AD, Tourette syndrome, or PD [33,37]. Our knowledge is still limited regarding the role of miRNAs in mature neurons although they may contribute to the maintenance of synaptic plasticity [40]. This evidence was supported by the studies on *Dicer* knockdown mice in which miRNAs loss in mature neurons of the forebrain, induced an increase of learning and memory skills associated with an overexpression of plasticity-related genes [41].

### 3.4. MiRNAs Involved in the Pathogenesis of DN

Recent studies highlight that miRNAs play a role in the development of DN. On a speculative basis, miRNAs involved in retinopathy and nephropathy inflammation also affect neuropathic inflammation, i.e., miRNAs able to regulate nuclear factor κB (NF-κB) [42]. Moreover, the release of cytokines such as IL-1β, MCP (monocyte chemoattractant protein)-1 and TNF-α from neuropathic tissues following long-term hyperglycemic state, suggests the infiltration of M1 macrophages and the consequential involvement of miRNAs in this process [43]. Furthermore, miR-214 and miR-21 targeting PTEN (phosphatase and tensin homologue deleted on chromosome 10) in the kidney may also regulate inflammatory gene expression and signaling following NF-κB activation in DN [44]. Hyperglycemia often damages endothelial cells thus contributing to vascular complications. Several studies have highlighted the importance of VEGF and its alteration during the development of diabetic retinopathy [45]. Similarly, VEGF and its regulating miRNAs may be involved in the progression of DN and therefore may be attractive targets for treatment. In fact, a clinical trial using intramuscular VEGF gene delivery showed efficacy on pain perception in a significant number of subjects [46]. However, since VEGF treatment induces several side effects, the use of VEGF-associated miRNAs could be a valid therapeutic alternative to the VEGF gene delivery. TGF-β has been highlighted as an important mediator of neuronal function and growth through regulation of several miRNAs that could be involved in neuropathy and represent therapeutic targets [47].

### 3.5. MiRNAs Contribute in Animal Models of DN

**Figure 1:**
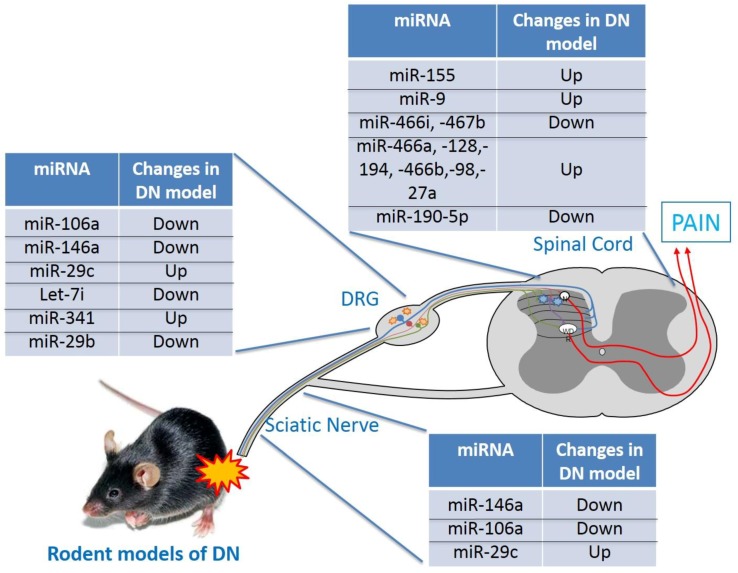
Schematic illustration of miRNAs involved in animal model of DN.

Both hyperglycemia and hypoxia are diabetes hallmarks, together with metabolic pathway alterations, may lead to complications such as DN [48]. Although the pathogenic mechanisms of DN remain to be fully elucidated, a correlation between specific miRNAs and its onset and development was suggested (Figure 1).

As regard DPN was induced in rats fed with a high-fat/sugar diet for 6 weeks and subjected to intraperitoneal injection of streptozotocin (STZ) followed by further 6 weeks of high-sugar feeding [49]. Thus, 6 weeks of uncontrolled diabetes reduced miR-146a expression in the sciatic nerve of DPN rats. Furthermore, the high-sugar diet prolonged for additional 6 weeks induced a significant reduction of both motor and sensory nerve conduction velocity. These changes were not accompanied by NF-κB downregulation in the sciatic nerve indicating a possible link between miR-146a and this pro-inflammatory transcription factor [49]. Indeed miR-146a gene presents a binding site for NF-κB that induces its expression during inflammation-related hyperglycemia [50]. Conversely, miR-146a inhibits post-transcriptionally the interleukin (IL)-1 receptor-associated kinase 1 (IRAK1) and TNF-receptor-associated factor 6 (TRAF6) that mediates NF-κB activation [51]. Accordingly, hyperglycemia might activate NF-κB causing miR-146a upregulation. This leads to IRAK1 and TRAF6 downregulation and subsequent NF-κB inhibition [49]. However, long-term hyperglycemia induces miR-146a downregulation in the sciatic nerve of DPN rats causing a loss of NF-κB inhibition, tissue damage, TNF-α, and IL-1β release [49]. Similar results were obtained when 20 weeks old *db*/*db* mice were used as model of T2D [52]. In this study, downregulation of miR-146 in the sciatic nerve compared to the control group was observed meanwhile the miR-146 systemic administration mimics suppressed inflammatory genes in the sciatic nerve through NF-κB inhibition and improved neurological functions [52]. Moreover, Liu XS et al. [52] confirmed that IRAK 1 and TRAF6 are putative targets of miR-146a but in addition they used bioinformatics to predict ADAMST3 (a disintegrin and metalloproteinase with thrombospondin motifs 3) as additional target gene confirming its role in the inflammatory processes [53]. Therefore, miR-146a mimics significantly improved sciatic nerve neurovascular functions and reduced inflammation suggesting its therapeutic application for the DPN treatment [52]. In the same animal model, hyperglycemia induced miR-146a downregulation and a concomitant increase of IRAK1 and TRAF6 in dorsal root ganglia (DRG) neurons. These effects were reverted by treatment with sildenafil [54]. Conversely, miR-146a and NF-κB expressions were found increased in the sciatic nerve after 2 months of uncontrolled diabetes induced in rats by a single intraperitoneal injection of nicotinamide followed by STZ [55]. Furthermore, TRAF6 mRNA levels in the sciatic nerve did not decrease in proportion to miR-146a overexpression; downregulation of IRAK1 mRNA was not significant [55]. Therefore, in these experimental conditions, miR-146a upregulation was unable to inhibit NF-κB through IRAK1 and TRAF6 downregulation. However, the levels of pro-inflammatory cytokines were significantly increased in the sciatic nerve of diabetic rats compared to control group, and the reaction time in response to the tail immersion and the hot plate tests were significantly decreased [55]. The reasons for this discrepancy compared with other studies may depend on the different DPN experimental models. In fact, 2 months post-injection of streptozotocin is enough time to establish a PN accompanied by upregulation of pro-inflammatory mediators such as NF-κB and its regulated cytokines. In this phase, the NF-κB-miR-146a negative feedback loop may be deregulated and the NF-κB upregulation induced by a hyperglycemic state leads to an increase of miR-146a. Conversely, prolonged exposure to hyperglycemia (more than 2 months) is responsible for miR-146a downregulation and uncontrolled NF-κB activation. Therefore, a steady state for miR-146a levels in the sciatic nerve is important to avoid hyperactivation of inflammatory pathways leading to nerve damage and DN.

Other miRNAs have been involved the development of DN. For example, in diabetic mice affected by PN, miR-106a reduces the oxidative/nitrosative stress induced by hyperglycemia in DRG and sciatic nerve improving both sensor and motor nerve conduction velocity [56]. Furthermore, the authors suggested that miR-106a might exert these effects by targeting the 12/15 lipoxygenase [56]. In another study performed on diabetic rats, the prophylactic treatment with *L*-arginine or ibuprofen or their combination reduced miR-155 expression and the levels of nitric oxide in the spinal cord decreasing mechanical allodynia [57]. In the same diabetes model of PN, miR-9 levels were found increased in the dorsal horn (DH) of spinal cord of neuropathic rats [58]. This upregulation was positively correlated to the expression of calcium homeostasis modulator 1 (CALHM1) even if TargetScan analysis did not confirm this gene as potential target of miR-9. However, miR-9 could contribute to the DPN by indirectly increasing CALHM1 expression and is a mechanism that still needs further investigation [58]. The upregulation of CALHM1 in the dorsal horn neurons induced the expression of purinergic receptor P2X, a ligand-gated ion channel 7 (P2X7) receptor that is expressed in various cells of nervous system and mediates ATP release and calcium concentration regulation in neuropathic pain. Therefore, the authors suggested that P2X7 receptor overexpression mediated by miR-9 upregulation sustained the mechanical hypersensitivity in diabetic rats [58].

The potential role of miRNAs in the regulation of DN was further confirmed by a microarray analysis of lumbar dorsal horn of spinal cords isolated from diabetic mice in which neuropathy was confirmed by the reduction of paw withdrawal thresholds [59]. In this study, miR-466i and miR-467b were downregulated in the lumbar dorsal horns of neuropathic mice meanwhile miR-466a, miR-128, miR-194, miR-466b, miR-98, miR-27a, and miR-194 were upregulated. All these miRNAs have been shown to target both pro- and anti-inflammatory cytokines that may contribute to the DN [59].

In another microarray analysis of miRNAs in DRG tissues of DPN rats, 37 miRNAs were differentially expressed in the diabetic group. Specifically, 15 and 22 miRNAs were upregulated or downregulated, respectively [60]. In particular, using a miRanda database, 10,011 predicted target genes were found from the 37 dysregulated miRNAs; a miRNA-gene-network analysis revealed that among these miRNAs, miR-330-5p, miR-17-1- 3p, and miR-346 had a high degree of correlation [60]. Meanwhile, podocalyxin-like (Podxl, inhibiting cell-cell adhesion) and homeobox A1 (Hoxa1, sequence specific transcription factor) were the most common target mRNAs with the highest degrees of connectivity [60].

Furthermore, by using the KEGG database, 29 enriched signaling pathways including metabolic, HIF-1, calcium signaling, PI3K-Akt, as well as p53-signaling pathways were discovered. The top downregulated pathways included the ECM-receptor interaction, focal adhesion, and biosynthesis of unsaturated fatty acids, while the top upregulated pathways included HIF-1 signaling pathway, neuroactive ligand-receptor interaction and metabolic pathways.

In streptozotocin-induced model of DPN, miR-190-5p downregulation was detected in the lumbar dorsal horn accompanied by an increase of both gene and protein of solute carrier family 17A6 (SLC17A6) also known as VGLUT2 [61]. This transporter is responsible of glutamate packaging into synaptic vesicles and plays a key role in the fast-excitatory synaptic transmission in the vertebrate nervous system (reviewed (rev) in [61]). SLC17A6 is significantly up-regulated in rat DRG and spinal cord following nerve injury [62]. In fact, deletion of SLC17A6 in related nociceptors reduces acute heat, mechanical and chemical pain responsiveness in neuropathic pain models (rev in [61]). Therefore, SLC17A6 was shown to be direct target of miR-190-5p and subarachnoid injections of a SLC17A6 inhibitor or a Lentivirus carrying miR-190-5p mimic were able to reduce the mechanical hypersensitivity and the levels of IL-6 and IL-1β in lumbar spinal dorsal horn samples [61].

MicroRNAs are considered important players of intercellular communication and found encapsulated into extracellular vesicles (EVs) including exosomes released by all living cells [63,64,65]. On this regard exosomes derived from Schwann cells exposed to high concentration of glucose were found enriched of miR-28, -31a and -130a. In vitro, treatment of DRG distal axons with high glucose (HG) exosomes resulted in the reduction of axonal growth, associated with elevation of these miRNAs and reduction in axons of their DNA target proteins methyltransferase-3a (NDNMT3A), NUMB (an endocytic adaptor protein), synaptosome associated protein 25 (SNAP25), and growth-associated protein-43 (GAP43) [66]. This suggests the involvement of specific miRNAs in the regulation of genes mediating distal axonal damage under diabetic conditions. Furthermore, administration of HG exosomes to sciatic nerves of diabetic 7-week-old *db*/*db* mice promoted occurrence of PN characterized by impairment of nerve conduction velocity and induction of mechanic and thermal hypoesthesia. This was associated with substantial decreases in intra-epidermal nerve fibers confirming a functional role of exosomes derived from HG-stimulated Schwann cells in mediating DPN development [66]. In order to elucidate the molecular mechanisms underpinning the hyperglycemia-mediated distal axonal damage of peripheral nerves, another study showed that miR-29c was upregulated in DRG neurons, sciatic nerve and foot pad tissues of diabetic mice [66]. A putative target of miR-29c was identified, i.e., the *PRKCI* gene that encodes for the isoform iota of PKC related to the regulation of axonal growth [67]. In their study, concomitantly with miR-29s upregulation, levels of PRKCI protein were reduced in DRG neurons and sciatic nerve. These effects resulted in the suppression of axonal growth of DRG neurons as demonstrated by in vitro experiments [66]. Therefore, miR-29c is a negative regulator of axonal growth of DRG neurons by targeting PRKCI under hyperglycemia, confirming the involvement of miRNAs in the molecular mechanisms underlying hyperglycemia-induced axonal damage [66].

miR-29b was also related to the axonal growth and protection of mature neurons against apoptosis induced by diverse insults [68]. miR-29b levels were downregulated in DRG primary sensory neurons of diabetic rats [69]. This downregulation worsened in time dependent manner, associated with higher apoptosis rate and axonal swelling [69]. Furthermore, the in vitro transfection of DRG neurons from diabetic rats with a miR-29b mimic stimulated axon regeneration and inhibited neuron degeneration-related genes expression [69]. SMAD family member 3 has been already reported as potential target of miR-29b and its inhibition ameliorated diabetic nephropathy [70]. Consistently, a Smad3 inactivation was observed after miR-29b restoration in DN [69].

Let-7i and miR-341 have been identified as miRNAs potentially involved in the progression of PN in diabetic mice [71]. A microarray profiling of miRNA expressed in DRG sensory neurons revealed significant changes in diabetic mice with downregulation of let-7i and increase of miR-341 [71]. Furthermore, to counteract this variation in miRNA levels, a let-7i mimic or a miR-341 inhibitor were delivered in two independent experiments into CNS by intranasal injection. Both approaches independently improved electrophysiological, structural, and behavioral changes without altering hyperglycemia [71]. A trophic action for miRNA let-7i was shown in in vitro experiments on dissociated adult sensory neurons exposed to it. Let-7i is predicted to target at least 46 apoptotic cell death pathway mRNAs, 42 cardiovascular and diabetes-related mRNAs, 84 growth, 80 inflammation-related pathway mRNAs, 21 metabolism and diabetes pathway mRNAs, and 59 neurotransmitter and nervous system mRNAs (ingenuity pathway analysis) [71]. Therefore, the authors concluded that central manipulation of DRG neurons is effective in altering the behavior of the entire neuronal tree, including its distal terminals; these data indicate a potential new, non-viral approach to target CNS or peripheral nervous system gene function [71] (Table 1).

### 3.6. Human Studies of MicroRNAs in DN

Several clinical studies show that specific miRNAs alterations may contribute to DN progression in patients and may represent a valid diagnostic tool for this complication.

Single nucleotide polymorphisms (SNPs) of miR-128a, miR-146a, and miR-27a were analyzed in order to correlate potential variations to the risk of developing peripheral or autonomic neuropathies in T2D [72]. MiR-128a rs11888095 was significantly correlated to a higher risk of developing DPN. Similarly, miR-146a variation rs2910164 was associated to a lower risk of DPN and protection in respect to early cardiovascular autonomic neuropathy (CAN). Conversely, miR-27a rs895819 SNP was linked to a higher susceptibility of developing early CAN in patients. T2D patients carrying the miR-499a rs3746444 SNP have higher predisposition to develop severe CAN [73].

MiR-199a-3p levels were increased in plasma and lower limb skin samples of diabetic patients affected by PN and with family history of diabetes [74]. Further upregulation of miR-199a-3p could act as a pro-coagulating factor in DN by targeting the extracellular serine protease inhibitor E2 (SERPINE2), contributing to the tissue vascular damage associated with PN. These studies suggest that miRNAs may be involved in the pathogenesis and progression of DN, and may serve as both biomarkers in early diagnosis and therapeutic target.

A total of 749 differentially expressed genes (DEGs) between non-progressing and progressing DN nerve samples, including 370 upregulated DEGs and 379 downregulated DEGs, was screened [75]. Using miR2Disease database, three specific miRNAs were associated with diabetes: miR-377, miR-216a and miR-217. The study predicted 1052 target genes of miR-377, 962 of miR-261a, and 1025 of miR-217 [75]. Inflammation was the most significantly represented biological process [75]. Furthermore, the Kyoto Encyclopedia of Genes and Genomes enrichment analysis revealed that peroxisome proliferator-activated receptor (PPAR) and adenosine monophosphate (AMP)-activated protein kinase (AMPK) signaling pathways were significantly regulated and enriched with PPAR gamma (PPARG), stearoyl-CoA desaturase (SCD), cluster of differentiation 36 (CD36), and phosphoenolpyruvate carboxykinase 1 (PCK1). This suggests their involvement in DN progression as possible biomarkers and potential therapeutic targets [75] (Table 2).

## 4. Conclusions

Neuropathy is considered the worst and most common complication of diabetes leading to greatest morbidity and mortality, more hospitalizations, therefore huge economic impact for diabetes care. About 50%–70% of non-traumatic amputations are due to DN [17]. This highlights the importance of early and accurate diagnosis for prevention and urgency to find novel therapeutic targets.

Since their initial discovery, miRNAs have rapidly gained attention due to their role of post-transcriptional gene regulators with effect on several developmental, physiological, and pathophysiological processes where they silence genes and modulate the expression of different proteins [25]. The role of miRNAs in diabetes [33] and its complications [34] is the object of intensive investigations not only for their involvement in the pathogenesis, but also as potential disease biomarkers. Therefore, in this review we discussed the most recent literature about miRNAs involved in the development of DN and the mechanisms by which they could regulate its onset and progression. In particular, Table 3 summarizes the main pathways regulated by these miRNAs according to the Kyoto Encyclopedia of Genes and Genomes (KEGG) database. In fact, both animal and human studies performed in the last 10 years show an increasing attention of the scientific community to the link between miRNAs and DN. This aspect not only increases our knowledge about the pathogenic mechanisms but also provides insights on novel and potential therapeutic targets for the treatment or the prevention of this complication. In fact, the use of selective miRNAs mimics or antagomiRs could represent a potential therapeutic tool with the aim to restore or counteract the reduction or the increase, respectively, of miRNAs levels in the nervous system. Moreover, thanks to their stability in serum and their easy detection, miRNAs may also represent a novel diagnostic tool for several diseases including DN [29]. Therefore, the detection of specific circulating miRNAs may be considered as a predictive biomarker for this complication. However, more investigations are required in future to pinpoint a whole miRNAs signature of DN and investigate its effectiveness of miRNAs and limitation in clinical practice for the management of this complication.

## Figures and Tables

**Table 1:** miRNAs studied in animal models of diabetic neuropathy.

miRNA	DN Model	Changes in DN	Target (s)	Reference (s)
miR-146a	DPN induced by intraperitoneal injection of STZ in HFD-fed rats	Down	IRAK1; TRAF6	Feng Y et al., 2018 [49]
miR-146a	*Db*/*db* mice as model of T2D and DN	Down	IRAK1; TRAF6; ADAMTS3	Liu XS et al., 2017 [52]
miR-146a	*Db*/*db* mice as model of T2D and DN	Down	IRAK1; TRAF6	Wang L et al., 2014 [54]
miR-146a	Diabetes was induced in rats by a single-dose injection of nicotinammide followed by STZ	Up	IRAK1; TRAF6	Yousefzadeh N et al., 2015 [55]
miR-106a	DPN model was established in mice following single injection of STZ	Down	12/15-Lipoxygenase (12/15-LOX)	Wu Y et al., 2017 [56]
miR-155	DN was established in rats following injection of STZ	Up	ND	El Lithy GM et al., 2016 [57]
miR-9	DN was established in rats following injection of STZ	Up	CALHM1 is indirect target of miR-9	Liu W et al., 2016 [58]
miR-466i	DPN was induced in mice following injection of STZ	Down	IL-1β; TNF-α; IL-6	Gong Q et al., 2015 [59]
miR-467b		Down		
miR-466a		Up	IL-1β	
miR-128; miR-194; miR-466b; miR-98		Up	IL-1β	
miR-27a		Up	IL-10	
miR-194		Up	IL-13	
37 miRNAs were differently expressed in DRG	DN was established in rats following injection of STZ	15 miRNAs were upregulated whereas 22 were downregulated	Podx1 and Hoxa1 were the most common targets	Guo G et al., 2018 [60]
miR-190-5p	DPN model was established in mice following single injection of STZ	Down	SLC17A6	Yang D et al., 2017 [61]
miR-28, miR-31a and miR-130a	In vitro exosomes isolated from high glucose stimulated Swann Cells contained high levels of miRs-28, -31a, -130	Up	NDNMT3A NUMB, SNAP25 and GAP43	Jia L et al., 2018 [66]
miR-29b	DN was established in rats following injection of STZ	Down	TGF-β/Smad3	Zhang X et al., 2014 [69]
Let-7i and miR-341	DPN model was established in mice following single injection of STZ	Down	NF-kB	Cheng C et al., 2015 [71]

DPN: Diabetic Peripheral Neuropathy; STZ: Streptozotocin; T2D: Type 2 Diabetes; IRAK1: Interleukin-1 receptor associated kinase 1; TRAF6: TNF-receptor associated factor 6; DRG: Dorsal root ganglion; NO: Nitric oxide; DH: Dorsal horn; ADAMTS3: A disintegrin and metalloproteinase with thrombospondin motifs 3; SLC17A6: Solute carrier family 17A6; CALHM1: Calcium homeostasis modulator 1; Podx1: Podocalyxin; Hoxa1: Homebox A1; NDNMT3A: DNA methyltransferase-3a; NUMB: an endocytic adaptor protein; SNAP25: Synaptosome associated protein 25; GAP43: Growth-associated protein-43; TGF-β: Transforming growth factor-β; Smad3: SMAD family member 3.

**Table 2:** MiRNAs studied in human diabetic neuropathy.

MiRNA	Target (s)	Changes in DN Patients	Clinical Manifestations	Reference
miR-499a; -128a; -146a; -27a	ND	Polymorphisms in the miRNA gene sequence	High incidence of developing CAN and DPN in T2D	Ciccacci C et al. [72,73]
miR-199a-3p	SerpinE2	Up in skin biopsies	Promote coagulation in peripheral skin circulation in T2D	Li YB et al., 2017 [74]
miR-216a	ANXA9	Up in nerve tissue samples	Positive association with progressing DN	Li YB et al., 2016 [75]
	ANGPTL4			
	CHI3L2			
miR-217	ADRBK2			
	CSN1S1			
	GALM			
miR-377	EN1			
	GAP43			
	FAM89A			

CAN: Cardiovascular autonomic neuropathy; SerpinE2: Serine protease inhibitor E2; ANXA9: Annexin A9; ANGPTL4: Angiopoietin-like 4; CHI3L2: Chitinase 3-Like Protein 2; ADRBK2: Beta-adrenergic receptor kinase 2; CSN1S1: Gene encoding for Alpha-S1-casein; GALM: Gene encoding for Aldose 1-epimerase; EN1: Gene encoding for homeobox protein engrailed-1; GAP43: Growth associated protein 43; FAM89A: Family with sequence similarity 89 member A.

**Table 3:** miRNAs involved in preclinical and clinical studies of diabetic neuropathy and the relative most regulated pathways.

MiRNA	Target Genes	Most Regulated Pathways	KEGG Pathway
miR-146a	IRAK1	NF-kB signaling pathway	mmu04064
		Toll-like receptor signaling pathway	mmu04620
		MAPK signaling pathway	mmu04010
		Neurotrophin signaling pathway	mmu04722
miR-146a	TRAF6	NF-kB signaling pathway	mmu04064
		Toll-like receptor signaling pathway	mmu04620
		Neurotrophin signaling pathway	mmu04722
		IL-17 signaling pathway	mmu04657
		NOD-like receptor signaling pathway	mmu04621
		MAPK signaling pathway	mmu04010
		Ubiquitin mediated proteolysis	mmu04120
		RIG-I-like receptor signaling	mmu04622
		Endocytosis	mmu04144
miR-106a	12/15-Lipoxygenase (12/15-LOX)	Arachidonic acid metabolism	mmu00590
miR-190-5p	SLC17A6	Synaptic vesicle cycle	mmu04721
		Glutamatergic synapse	mmu04724
miR-31a; miR-130a	NUMB; SNAP25	Notch signaling pathway	mmu04330
		Synaptic vesicle cycle	mmu04721
		Insulin secretion	mmu04911
miR-29b	Smad3	TGF-beta signaling pathway	mmu04350
		FoxO signaling pathway	mmu04068
		Wnt signaling pathway	mmu04310
		Th17 cell differentiation	mmu04659
		Cell cycle	mmu04110
miR-29c	PRKCI	Tight junction	mmu04530
		Rap1 signaling pathway	mmu04015
		Endocytosis	mmu04144
		Insulin signaling pathway	mmu04910
miR-466i; miR-467b	TNF-α; Il-6	NF-kB signaling pathway	mmu04064
		Apoptosis	mmu04210
		TNF signaling pathway	mmu04668
		HIF-1 signaling pathway	mmu04066
		PI3K-Akt signaling pathway	mmu04151
		Insulin resistance	mmu04931
		Toll-like receptor signaling pathway	mmu04620
		NOD-like receptor signaling pathway	mmu04621
miR-466a; miR-128; -194; -466b; -98	IL-1β	NOD-like receptor signaling pathway	mmu04621
		NF-kB signaling pathway	mmu04064
		IL-17 signaling pathway	mmu04657
		Th17 cell differentiation	mmu04659
		Toll-like receptor signaling pathway	mmu04620
		Inflammatory mediator regulation of TRP channels	mmu04750
miR-27; miR-194	IL-10; IL-13	FoxO signaling pathway	mmu04068
		TCR signaling pathway	mmu04660
		IL-17 signaling pathway	mmu04657
		Th1 and Th2 cell differentiation	mmu04658
miR-216a	ANGPTL4	PPAR signaling pathway	hsa03320
		Cholesterol metabolism	hsa04979
miR-217	ADRBK2 GALM	Glutamatergic synapse	hsa04724
		Glycolysis and gluconeogenesis	hsa00010
		Galactose metabolism	hsa00052

PRKCI: Isoform iota of Protein kinase C; MAPK: Mitogen-Activated Protein Kinase; FoxO: Forkhead Box O; HIF-1: Hypoxia Inducible Factor 1; PI3K: Phosphoinositide-3-kinase; ANGPTL4: Angiopoietin-like 4; ADRBK2: Beta-adrenergic receptor kinase 2; GALM: Gene encoding for Aldose 1-epimerase; PPAR: Peroxisome proliferator-activated receptor.

## Abbreviations

miRNAs	microRNAs
DM	Diabetes mellitus
T1D	Type 1 diabetes
HLA	Human leukocyte antigen
T2D	Type 2 diabetes
IR	Insulin resistance
ADA	American Diabetes Association
ED	Endothelial dysfunction
CVD	Cardiovascular diseases
PN	Peripheral neuropathy
NO	Nitric oxide
TGF	Transforming growth factor
DN	Diabetic neuropathy
DSPN	Distal symmetric polyneuropathy
DPN	Diabetic peripheral neuropathy
STZ	Streptozotocin
miRs	microRNAs
RNAi	RNA interference
AD	Alzheimer’s disease
PD	Parkinson’s disease
NF-κB	Nuclear factor κB
MCP	Monocyte chemoattractant protein
PTEN	Phosphatase and tensin homologue
VEGF	Vascular endothelial growth factor
IL	Interleukin 2
IKAK 1	Interleukin-1 receptor-associated kinase 1
TRAF6	Tumor necrosis factor receptor-associated kinase 1
ADAMTS3	A disintegrin and metalloproteinase with thrombospondin motifs 3
12/15-LOX	12/15 lipoxygenase
DRG	Dorsal root ganglia
DH	Dorsal horn
CALHM1	Calcium homeostasis modulator 1
P2X7	Purinergic receptor P2X ligand gated ion channel 7
Podxl	Podocalyxin-like
Hoxa1	Homeobox A1
SLC17A6	Solute carrier family 17A6
VGLUT2	Vesicular-glutamate transporter 2
rev	Reviewed
EVs	Extracellular vesicles
NDNMT3A	DNA target proteins methyltransferase-3a
NUMB	Endocytic adaptor protein
SNAP25	Synaptosome associated protein 25
GAP43	Growth-associated protein-43
HG	High glucose
PRKCI	Protein kinase C iota
SMAD	Small mother against decapentaplegic
CNS	Central nervous system
SNPs	Single nucleotide polymorphisms
CAN	Cardiovascular autonomic neuropathy
SERPINE2	Serine protease inhibitor E2
DEGs	Differentially expressed genes
PPAR	Peroxisome proliferator-activated receptor
AMP	Adenosine monophosphate
AMPK	AMP-activated protein kinase
PPARG	PPAR gamma
SCD	Stearoyl-CoA desaturase
CD36	Cluster of differentiation 36
PCK1	Phosphoenolpyruvate carboxykinase 1
ANXA9	Annexin A9
ANGPTL4	Angiopoietin like 4
CHI3L2	Chitinase 3 Like 2
ADRBK2	Adrenoceptor Beta 2
CSN1S1	Casein Alpha S1
GALM	Galactose mutarotase
EN1	Homeobox protein engrailed-1
FAM89A	Family with sequence similarity 89 member A
